# Making a Difference in PE Lessons: Using a Low Organized Games Approach to Teach Fundamental Motor Skills in China

**DOI:** 10.3390/ijerph16234618

**Published:** 2019-11-21

**Authors:** Li Zhang, Peggy Cheung

**Affiliations:** 1 School of Physical Education, Chongqing University, Chongqing 401331, China; zhangli0305@cqu.edu.cn; 2 Department of Health and Physical Education, The Education University of Hong Kong, Hong Kong, China

**Keywords:** fundamental motor skills, low organized game, physical activity, children, physical education

## Abstract

Background: Fundamental motor skills (FMS) is the foundational movement for children’s physical development. Physical Education (PE) lessons provide a unique opportunity for children to acquire these skills. The purposes of this study were to: (1) to describe the FMS performance of primary school children in China; and (2) to examine the effect of a PE-based intervention on children’s FMS. Methods: The 12-week PE-based intervention was conducted using a low organized games (LOG) approach. Children’s FMS was measured by the Test of Gross Motor Development-2 (TGMD-2). Participants were 560 children aged between 6–9 years old (grade one to three) from two primary schools in Chongqing, China. They were grouped into intervention group (with LOG program PE; *n* = 282) or control group (with regular PE; *n* = 278). Results: The results revealed significant interaction effect between group and grade on the locomotor skill score changes (F_(1,554)_ = 22.31, *p* < 0.000), and object control score change (F_(1,554)_ = 627.1, *p* < 0.000). There was no significant interaction effect between the intervention group and gender on locomotor skill (F_(1,554)_ = 1.49, *p* = 0.223) and object control skill significant (F_(1,554)_ = 743, *p* = 0.389). Conclusions: The present study supported the application of LOG approach in PE lesson as an effective intervention to enhance children’s FMS in China.

## 1. Introduction

Fundamental motor skills (FMS) are defined as an organized series of basic movements involving the combination of movement patterns of two or more body segments [1]. FMS are considered foundational to participation in games, sports and other physical activities, representing a potentially important factor affecting children’s participation in physical activity (PA) [2]. Mastering FMS helps children control their bodies and develop complex skills in physical sports and game activities [3], encouraging maximal participation in PA and spontaneous enjoyment, and allowing young people to both understand their competence and improve their confidence in maintaining an active lifestyle [4].

A previous study reported positive relationships between PA, FMS development and fitness [5]. Improving FMS is considered to be relevant to positive health-related outcomes as well as increased PA participation [6]. Despite the importance of FMS, mastery of FMS among children in most countries are not positive. One study of 9-to-12-year-old children in the United States reported that only 50% of students were proficient at dribbling and throwing a basketball, while a study conducted in Australia revealed that approximately 40% of children (aged between 4 and 10 years old) can only master one FMS [7].

A common misconception regarding FMS is that children can naturally acquire FMS as they mature. However, there is evidence that systematic FMS instruction must be provided to children in the early childhood years, enabling them to learn, practice and acquire FMS in childhood [8]. The reasons that children failed to master FMS are claimed to include a lack of opportunity, encouragement, and guidance in physical activity practice [8]. Physical Education (PE) lessons provide opportunities for PA among children, representing a principal source of acquiring and mastering FMS [9]. PE lessons provide an important setting for teaching children FMS by offering supportive environments [10] and opportunities for practice [11]. PE-based interventions have been reported to be efficacious for improving children’s motor skill proficiency [12]. In addition, the enhanced motor skills component in the PE curriculum has been related to improvements in gross motor development among first-grade [13] and fourth and fifth grade children [14]. Children’s mastery of FMS is involved in the PE curricula in Portugal, China and the United States, with a structured PE curriculum related to FMS associated with a higher proportion of children achieving FMS mastery [15]. However, the previous study in China have only been conducted in Hong Kong, which has a different PE curriculum compared with other cities in China. Thus, the benefits of PE-based intervention on children’s FMS in China remain unclear.

Teaching of FMS is included in the PE curriculum in many countries, including the United States, Australia, the United Kingdom, Hong Kong, and mainland China [7]. With differences in cultural background and education system, pedagogical approaches to teach FMS during PE lessons vary widely. In China, PE is a compulsory part of the national curriculum set by the Ministry of Education (MOE) of the People’s Republic of China. School PE lesson focuses heavily on sporting skills, and the main teaching content of the PE curriculum involves competitive sports, such as track and field, basketball, football, volleyball and gymnastics [16]. Despite growing concern regarding the importance of FMS development for children’s health, teaching of FMS in PE lessons in China is not a usual practice. In a review of PE-based interventions with FMS-focused studies, various remarks were highlighted, including detailed theoretical and pedagogical approaches have not been clearly elucidated [12]. It is also observed that direct instruction teaching methods and providing a mastery motivational climate are pedagogical approaches reported to be effective in PE-based interventions [13,17]. Time constraints are another factor that must be considered in FMS teaching in PE lessons, and the instruction time required for FMS teaching may mean that students are not provided with a sufficient amount of exercise in a PE lesson [18]. In regards to the concerns on teaching FMS during PE lessons, the current study focused to address the research question: can the FMS ability of children in China be improved by making use of a PE-based intervention?

The present study implemented a PE-based intervention that aimed to enhance children’s FMS ability. The intervention design was based on dynamic systems theory (DST) [19], highlighting the importance of interactive effects among individual, task and environmental constraints on children’s FMS development [20]. Low-organized games (LOG) was adopted as the pedagogical approach for the teaching, which could be applied to focus on the desired objectives of the lesson [21]. LOG constitutes a form of PA that requires simple rules to conduct activities and provides participants with opportunities to express, explore, and discover various aspects of their lives and the lives of others [22]. A previous intervention study based on LOG programs elicited a significant improvement of FMS among 6-to-10-year-old children after 11 weeks of engagement in the program [23]. However, with the small sample size (*n* = 40) in this study, the intervention effect was not clear. The purpose of the current study are: 1) to describe the FMS ability of primary school children in China; and 2) to examine the effect of a PE-based intervention on children’s FMS ability. 

## 2. Materials and Methods

The present study used a quasi-experimental design to examine the effects of a 12-week PE-based program on children’s FMS ability. Data were collected from two elementary schools in Chongqing, China. Ethical approval for using human subjects was obtained from the ethical committee (Ref. no. 2015–2016–0362) of the research university and consent for participation was confirmed by the participating schools and children’s parents.

### 2.1. Participants

Participants were elementary school children (grade 1 to 3) studying in an urban district in Chongqing, China. Among all thirty-four government-funded elementary schools in the district, two schools with comparable numbers of teachers and students were recruited to participate in the study, through convenience sampling. Children in the lower forms (i.e., grades 1 to 3) at each school were recruited as participants. The six selected classes in each school were randomly assigned into the intervention group (with LOG program PE; *n* = 282) or the control group (with regular PE; *n* = 278). A total of 560 children (mean age = 7.54, SD = 0.94; 57.3% boys and 42.7% girls) were involved in the study. Before the first teaching session, children’s anthropometric data were collected, including body height and weight, the BMI Z-scores were generated using the World Health Organization (WHO) growth standards. Descriptive information about the participants (i.e., age, height, weight and BMI Z-scores) is shown in Table 1.

### 2.2. Intervention Procedure and Content

The study was conducted for twelve weeks in the PE lessons by the PE teacher of each school. Each group of students attended the 40-min PE lesson twice a week during the regular PE lesson schedule. Before the study, two 2-h training sessions were arranged for PE teachers in the intervention group. The training session included the introduction of the approach, content and practice of the LOG program. A briefing session for the children was conducted prior to the intervention, when the baseline measures for the FMS score and anthropometric measures were obtained. After the 12-week intervention, all participants were given a measurement on FMS, following the same procedure as the pre-intervention stage. 

#### 2.2.1. Intervention Group with LOG Content

The intervention content was designed to focus on the core concepts of DST, with the emphasis on the development constraints of motor skills in children, including individual, task and environmental constraints. For example, regarding individual constraints, the program design activity (e.g., throwing and kicking) with variations in strength requirements. Concerning the task constraints imposed by the equipment, balls were made of a cushion-like material to facilitate the actions of grasping and two-handed catching. Regarding environmental constraints, the program included group activity for skill practice and peer interaction. Each PE lesson was conducted with three components: (a) 5-min warm-up games, (b) 30-min of LOG tasks (FMS instruction and practice), and (c) 5-min cool down. The teaching content of each lesson include games that aims to train one of the 12 FMS, i.e., running, horizontal jumping, hopping, leaping, galloping, sliding, stationary dribbling, catching, kicking, striking a stationary ball, overhand throwing, and underhand rolling. 

#### 2.2.2. Control Group with Regular PE Lessons

Participants in the control group participated in regular PE lessons following the standard school curriculum. A regular PE lesson included three parts: (a) 5-min warm-up, (b) 30-min theme-based activity (e.g., basketball, volleyball, gymnastics, etc.), and (c) 5-min cool down.

### 2.3. Measures

Children’s FMS ability was measured using the Test of Gross Motor Development 2nd edition (TGMD-2) [24]. TGMD-2 consisted of subtests to assess: (1) locomotor skills, including running, horizontal jumping, hopping, leaping, galloping and sliding; and (2) object control skills, including, stationary dribbling, catching, kicking, striking a stationary ball, overhand throwing and underhand rolling. Scoring of the TGMD-2 was according to the fulfillment of the 3–5 performance criteria set for each skill. The total scores were summed up with a possible range between 0 and 96 points. High scores indicated better FMS ability, and vice versa. Standard scores for the sub-items (locomotor and object control skills) were calculated from the raw scores and converted to gross motor quotient (GMQ) scores. The GMQ was adopted for classifying the FMS performance of each child, from very poor to very superior [24]. The TGMD-2 is reported to be a reliable (0.86–0.92) and valid (0.71–0.93) instrument for assessing FMS in children aged between 3 and 10 years old [24]. 

### 2.4. Data Analyses

Statistical analysis was performed using the SPSS software package, version 24.0 (IBM Corp., Armonk, NY, USA). Descriptive data (mean and standard deviations) of participants’ anthropometric measures and FMS scores (locomotor, object control and FMS score) at baseline were computed. Changes of FMS score (posttest−pretest) were analyzed using an analysis of variance (ANOVA) to evaluate whether significant changes in children’s FMS scores occurred over 12 weeks between the intervention and control groups, children of different genders (boys and girls) and children in different grades (grade 1, 2 and 3). The effect size of the intervention was reported using the Cohen’s standard method (d) at a significance level of *p* < 0.05, with 0.2, 0.5 and 0.8 denoting small, moderate and large effect sizes, respectively [25].

## 3. Results

### 3.1. Participants’ FMS Performance

The baseline measurement of children’s FMS score is shown in Table 2. Children’s FMS performance was recorded as: locomotor score (M = 28.60, SD = 6.27), object control score (M = 24.65, SD = 5.32) and total FMS score (M= 53.20, SD = 9.74). The FMS performance (GMQ: M = 66.43, SD = 8.95) was classified as very poor, according to the TGMD-2 norms in the United States [24]. Independent samples *t*-tests revealed non-significant differences in the total FMS score (F(_1, 558)_ = 0.584, *p* = 0.445) between the intervention group (M = 53.51, SD = 10.10) and control group (M = 52.88, SD = 9.37) at baseline.

### 3.2. Changes of FMS Performance with PE-Based Intervention among Different Grades

The PE-based intervention yielded a significant difference in the changes of FMS performance (locomotor skill and object control skill) between the intervention and control group among different grades (Table 3). For the changes of locomotor skill, the results revealed a significant interaction between the effects of group and grade on score changes (F_(1,554)_ =13.43, *p* < 0.001). A simple main effect (F_(1,554)_ =79.10, *p* < 0.001) indicated that the intervention group (mean = 8.70, SD = 9.40) exhibited greater score changes than the control group (M = 2.86, SD = 6.06). For changes in object control skill, the results revealed a significant interaction between the effects of group and grade on score changes (F_(1,554)_ = 22.31, *p* < 0.001). A simple main effect (F_(1,554)_ = 228.31, *p* < 0.001) revealed that the intervention group (M = 12.12, SD = 5.80) exhibited greater score changes than the control group (M = 5.30, SD = 4.81).

### 3.3. Changes of FMS Performance with PE-Based Intervention between Gender

The PE-based intervention did not exhibit a significant difference in the changes of FMS performance (locomotor skill and object control skill) between the intervention group and gender (Table 4). For changes in locomotor skill, the results revealed significant main effects of group (F_(1,554)_ = 77.88, *p* < 0.001) and gender (F_(1,554)_ = 5.35, *p* = 0.021). The interaction effect was non-significant (F_(1,554)_ = 1.46, *p* = 0.228). For the changes of object control skill, the results revealed a significant main effect on group (F_(1,554)_ = 224.58, *p* < 0.001). The main effect of gender (F_(1,554)_ = 0.138, *p* = 0.711) and the interaction effect were non-significant (F_(1,554)_ = 0.069, *p* = 0.793).

## 4. Discussion

The present study examined FMS performance among children in China, and investigated the effectiveness of a PE-based intervention on the improvement of FMS. The positive improvement elicited by the intervention supported the teaching FMS in PE lessons using a LOG approach. 

### 4.1. FMS Performance of Children in China

Data from the baseline measurement on children’s FMS ability revealed that children in China scored comparatively low using the measurement scale of TGMD. Generally, the below average on FMS performance was reported in elementary school children of different countries, such as Singapore [26] and Italy [27]. Despite there is an inclination of low competency of children’s FMS, the differences in PE curricula between countries are reported to be associated with the proportion of children that master FMS [15]. Countries with national PE curricula focused on developing a high level of FMS competency, such as the United States and the United Kingdom, treat FMS development as the principal goal of quality PE in primary schools [9]. In contrast, school-based PE in China emphasizes sport skills [16]. This may be one of the possible factors related to the performance of children’s FMS in China. In addition, the skills included in the TGMD assessment tool were proposed by the National Association for Sport and Physical Education based on the cultural context of the United States [15], representing another potential culture-related factor influencing children’s FMS performance. The measurement conducted in the present study provides important information supporting the urgency of developing tailored intervention programs for FMS among children in China.

### 4.2. Effect of PE-Based Intervention on Children’s FMS Performance

The present study showed a positive effect of a PE-based intervention on children’s FMS performance. To the best of our knowledge, this is one of the very few studies addressing the teaching and learning of FMS conducted in PE lessons in China, the intervention design and pedagogical approach warrants deliberation. The intervention of the present study made use of PE lesson to enhance children’s FMS performance. It was congruent to previous successful intervention with a PE-based design [18,28,29]. PE lesson provide structured learning environment and practice opportunities, which have been emphasized as an important part of developing children’s FMS [1]. In China, the MOE provides standardized teaching materials for the basic content of the PE curriculum. Children were having PE lesson in a structured modes of learning. However, it was observed that children spend a substantial amount of time standing in line during PE lessons [30]. Skills-based PE lessons in China leave limited time for children to learn or practice FMS. In contrast, the LOG approach in the current study required simple rules and most of the time was available for children to practice movement. For example, each teaching session included simple games in the warm up, main activity and cool down periods, maximizing children’s opportunities to perform FMS. Previous studies reported a positive result of the implementation of the LOG program during an afterschool program [23]. Given the time constraints and curriculum specificity in PE, programs using a LOG approach could provide an alternative method for enhancing children’s FMS during PE lessons. 

### 4.3. Age Difference in the Change in FMS Performance with PE-Based Intervention

The current findings revealed that children in different grades obtained different levels of gains in locomotor skill and object control skill. For locomotor skill, younger children (grade 1) exhibited a greater improvement than older children (grades 2 and 3) after receiving the PE-based intervention. For object control skill, younger children (grade 1 and 2) also exhibited greater improvement than older children (grade 3). Overall, children in lower age groups were found to exhibit greater improvements in FMS. Previous studies indicated an age-related association on children’s FMS performance. A positive association between increasing age and children’s FMS was reported in preschool children [31] and an age-related increase in motor performance across all ages was reported in elementary school children [32,33]. However, the intervention effect on children of different age groups was not available for comparison. 

In the current study, children of all grades received a new experience of motor skill learning. This new experience exerted a positive effect on children’s FMS performance. Following the phases of motor development [19], 6 to 7 years of age represents the fundamental movement stage, involving the acquisition of locomotion, stability and manipulation. In addition, children at this age are strongly influenced by the environment and the people that surround them [34]. It is possible that there are periods during the early childhood years in which certain skills are learned more efficiently than at other times. The current findings may provide a basis for future interventions designed for children of different ages. 

### 4.4. Gender Difference in the Change of FMS Performance with PE-Based Intervention

The present results did not reveal a significant gender difference in changes in FMS performance after the intervention, indicating that the intervention induced similar effects on FMS improvement for boys and girls. This finding is consistent with the results of previous studies reporting no gender differences in the effectiveness of intervention on overall FMS [33,35] or locomotor skills [36]. However, some previous studies reported that girls benefited more in object control skills through an intervention program [4,36]. Environmental constraints are one of the key factors affect children’ motor skill development [19]. Gender differences in motor skills at a young age are speculated to be more strongly influenced by environmental rather than biological factors [37]. Similarly, regarding the intervention effects of children of different genders, environmental factors are expected to play an important role. Children in the current study had comparable backgrounds regarding physical training. In the context of the current study, the task and environmental constraints for FMS learning were likely to be similar between genders. The teaching of PE in China is conducted in a co-ed format, meaning that both genders were exposed to the same experience of FMS training through the LOG program. This may explain the lack of a difference in FMS changes between boys and girls in the current study.

Given the positive findings from the PE-based intervention on children’s FMS in the current study, some limitations should be considered. First, the current study was designed to implement a PE-based FMS intervention in China, and the PE classes were conducted by regular PE teachers without controlling for teachers’ experience or educational background. Similarly, with minimal interruption to the class schedule, the teaching content of the control group was adhered to the school curriculum. Second, the data were all collected from two primary schools in one province in China. With the focus on the effect of the program, factors such as BMI and age was not used for direct analysis. Future studies should include samples from a wider geographic area, or be expanded to a multi-country analysis, in consideration of other demographic factors for analysis. Third, the post-intervention measurement of FMS may be most useful for examining the immediate effects of intervention. Future studies should extend the follow-up period to examine the long-term effects of the intervention.

## 5. Conclusions

The present study examined the effectiveness of a PE-based intervention designed to improve FMS mastery among children in China. The positive findings of the current study could serve as an update and supplement the PE curriculum for primary schools in China. It highlighted the importance of environmental factor on children’s acquisition of motor skills. In the setup of the PE curriculum plan in respective primary schools, the teaching of FMS should be included, in the consideration of current pedagogy and teaching content. 

## Figures and Tables

**Table 1:** Descriptive data of the participants by gender (*n* = 560).

Variables	Boys (*n* = 321)	Girls (*n* = 239)	Total (*n* = 560)
Age (years)	7.63 (0.95)	7.41 (0.92)	7.54 (0.94)
Height (cm)	126.43 (9.20)	124.26 (7.85)	125.50 (8.71)
Weight (kg)	26.88 (6.41)	25.17 (4.38)	26.15 (5.69)
BMI Z-scores	−0.22 (1.83)	−0.25 (1.88)	−0.20 (1.78)

Note: Standard deviations appear in parentheses after the mean values.

**Table 2:** Participant’s FMS performance at baseline by age (*n* = 560).

Age	Locomotor Skill Score	Object Control Skill Score	FMS Total Score	Gross Quotient Score (GMQ)
6	23.77 (4.74)	21.76 (3.03)	45.60 (5.91)	72.24 (5.65)
7	25.99 (5.21)	22.77 (4.24)	48.70 (7.25)	66.69 (8.02)
8	30.34 (5.90)	25.29 (5.30)	55.59 (9.14)	63.30 (9.20)
9	34.49 (3.71)	29.52 (5.19)	63.86 (6.19)	66.97 (8.03)
Average score	28.60 (6.27)	24.65 (5.32)	53.20 (9.74)	66.43 (8.59)

Note: Standard deviation appear in parentheses after mean.

**Table 3:** Changes on FMS score between intervention and control group by grade (*n* = 560).

Skill/Grade	FMS Score Change Mean (SD)		F	*p*	Cohen’s d
	Intervention Group	Control Group			
Locomotor skill			13.43	0.00 *	0.54
1	13.17 (11.09)	2.78 (2.88)			
2	7.57 (8.81)	3.24 (3.99)			
3	4.95 (4.99)	2.46 (9.95)			
Object control skill			22.31	0.00 *	0.63
1	13.95 (4.49)	4.16 (4.52)			
2	12.89 (5.74)	5.50 (4.54)			
3	8.95 (6.04)	6.42 (5.23)			
Total FMS			26.78	0.00 *	0.85
1	26.94 (12.68)	6.92 (5.13)			
2	20.48 (11.47)	8.88 (6.09)			
3	14.18 (7.99)	9.02 (11.28)			

Note. * *p* < 0.001.

**Table 4:** Changes on FMS score between intervention and control group by gender (*n* = 560).

Skill/Gender	FMS Score Change Mean (SD)		F	*p*	Cohen’s d
	Intervention Group	Control Group			
Locomotor skill			1.46	0.23	0.17
Boys	7.68 (9.35)	2.54 (6.50)			
Girls	10.05 (9.35)	3.29 (5.41)			
Object control skill			0.07	0.80	0.04
Boys	12.00 (5.93)	5.28 (4.88)			
Girls	12.29 (5.65)	5.33 (4.73)			
Total FMS			1.10	0.29	0.15
Boys	19.69 (12.03)	7.89 (7.83)			
Girls	22.38 (12.07)	8.75 (7.55)			
